# Supplementary material for: The heartbeat of the city
Source: PLoS One. 2021 Feb 24;16(2):e0246714. doi: 10.1371/journal.pone.0246714 (PMC7904221; doi:10.1371/journal.pone.0246714)
Supplement: S1 File — (PDF) [file pone.0246714.s001.pdf]

S1 File

1.1 Cleaning and processing the data

Open Access data available at `https://datos.cdmx.gob.mx/explore/dataset/carpetas-de-investigacion-pgj-de-la-ciudad-de-mexico` gives the location and time of more than one million crimes reported to the Investigative Police from Mexico City from 2016 and until July 2020, including reports of domestic violence (9.2% of the data), shoplifting (6.3%), fraud (5.8%) and others. Some types of crime do not identify urban activities at specific moments (such as fraud or forgery) whilst others are not urban on its nature (such as domestic violence). Therefore, only robberies with a present victim are kept, which gives us precision in terms of the time of the crime and its location. A few crimes are reported outside Mexico City, or are reported after 2016 but occurred before that year and are also filtered from the data (less than 1% of the data). Crimes reported after 31st March are filtered out, as it is possible that the quarantine in the city changed the temporal and spatial patterns of crime drastically. In total, 222,741 crimes are kept (21.8% of the reported data). Robbery of a person in the street or public transport represents the majority of crimes kept (48.4% of the kept crimes) and robbery of a business and stolen property the second type of crime analysed (35.4% of the kept crimes). Unfortunately, not all crimes are reported to the police. According to the Mexican Victimisation Survey ENVIPE, in Mexico City, only 6% of

crimes are reported [1], which induces many types of biases in the data. However, the time in which the crime happened should not alter significantly whether it gets reported to the police or not. Although we only have a small fraction of the crimes which are suffered in the city, we assume that the corresponding shape of the heartbeats is representative of the whole crimes.

The road accidents data is registered by the *Centro de Atención a Emergencias C5*, the Emergency Attention Centre from Mexico City at the time that a crash is reported to the 911 or observed by a police officer through the CCTV in the city or other (similar) emergency attention sources (including emergency buttons, an app and social media). Only confirmed reports of a crash are kept and the time of the report is considered as the time of the accident. In total, 939,361 crashes are kept, including collisions between drivers (all types of vehicles and motorcyclists) or between a driver and cyclists or pedestrians. Because of the attention required and the visibility of crashes, most of the serious and fatal accidents which happen in Mexico City should be included in our analysis and few missing crimes should not alter our results.

1.2 Choosing bandwidth

The data, applying some frequently used rule of thumb (for instance, Silverman's rule [2]), is not capable of differentiating hours of a day and so smaller bandwidths are used. However, caution is needed with very small bandwidths. The data used here comes from crimes reported to the police in which the victim recalls the time in which they suffered the crime and report it. From all the crimes in our dataset which were reported in the 20-minute interval between 11:50 and 12:10, nearly 85% of them were reported to have happened exactly at 12:00, which is what the victim identifies as the moment of their crime. Therefore, although in theory crime can happen at every moment in time (and so we are dealing with a continuous variable) most of the crimes are reported at even hours (40% of crimes), with a smaller frequency at half hours or 15-minute breaks (33% of the crimes, considering it has three times the chances of a crime being reported, compared to even hours), and with a very low frequency at different 5-minute breaks, such as 12:05 or 12:10 (20% of the crimes, considering it has two times the chances of a crime being reported, compared to even hours or 15-minute breaks). Almost never crimes are reported at any other moments, such as 12:04 (7% of the crimes). The bandwidth picked smooths crime such that high peaks at even hours or 15-minute breaks are not observed and the same bandwidth was used for crashes.

1.3 Are the heartbeats random?

We construct a test to check whether the observed heartbeat of a region is statistically different from others, or to what can be obtained by randomness. To do it so, we consider a pair of crime heartbeats, H_1 and H_2, and we start with two tiles with a high correlation (0.9606). We try to obtain the same heartbeat as one of the regions H_2 by resampling the observed data from Mexico City. In the test, H_2 has 2,891 crimes, so we sample without replacement 2,891 crimes, construct their simulated heartbeat (H_s) and compute its correlation with H_1. By sampling the actual data, we are considering what a heartbeat could be with the same number of crimes and the underlying time distribution of crimes in the city, but without this sample being the group of crimes which gives H_2. We simulate 500 times following the same procedure and obtain a 95% interval, to get rid of potential outliers. The results show that a correlation as high as 0.9606, as observed between H_1 and H_2 cannot be obtained by sampling crimes (S1 Fig), as the 95% interval is between 0.77 and 0.86.

For the crashes heartbeats, a similar procedure by picking two regions with a high correlation (0.9608) and by sampling 9,987 crashes, as there are in one of the two tiles,

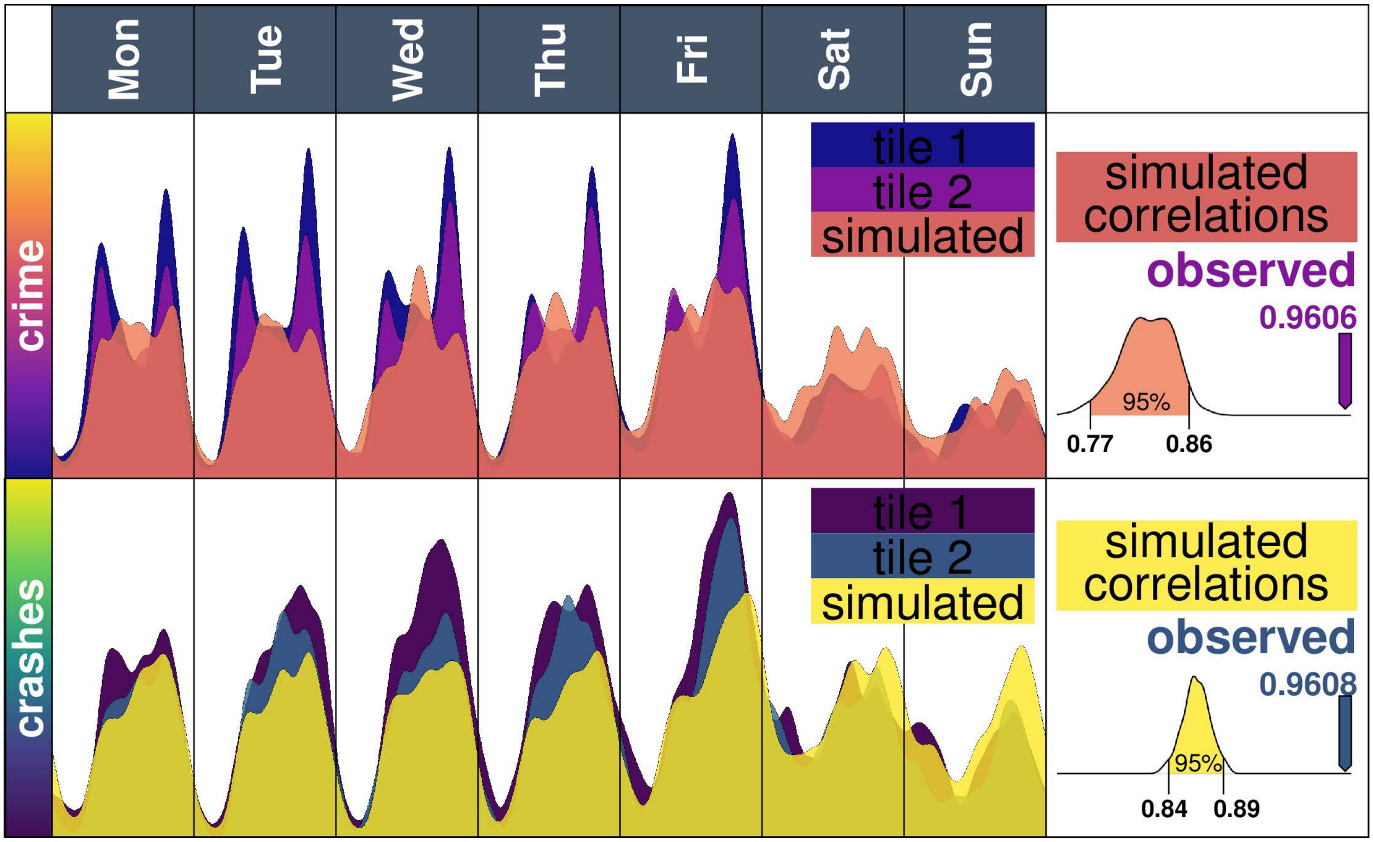

Mon
Tue
Wed
Thu
Fri
Sat
Sun
crime
crashes
tile 1
tile 2
simulated
simulated correlations
observed
0.9606
95%
0.77
0.86
tile 1
tile 2
simulated
simulated correlations
observed
0.9608
95%
0.84
0.89

S1 Fig. Testing a null hypothesis of random heartbeats. The top panel shows two highly correlated heartbeats from two distinct tiles in Mexico City and a simulated crime heartbeat, obtained by resampling crime data. The simulated heartbeats produce simulated correlations, which we can then use to produce the (0.77, 0.86) interval, on the right-hand side, which contains 95% of the simulated correlations. Therefore, the test helps reject the null hypothesis of randomness. In the case of crashes (bottom panel), a similar method helps us reject the null hypothesis.

allows simulating crashes heartbeats with the underlying time distribution of crashes in the city. Results show that a correlation between 0.84 and 0.89 is inside a 95% interval and therefore, we can also reject a null hypothesis of randomness in the crashes heartbeats (S1 Fig).

1.4 More or less tiles?

Different distance thresholds τ are tested. With a smaller distance, more tiles are obtained. With a smaller τ, tiles are divided and often crime and crashes exhibit a higher level of concentration on a smaller scale (S2 Fig).

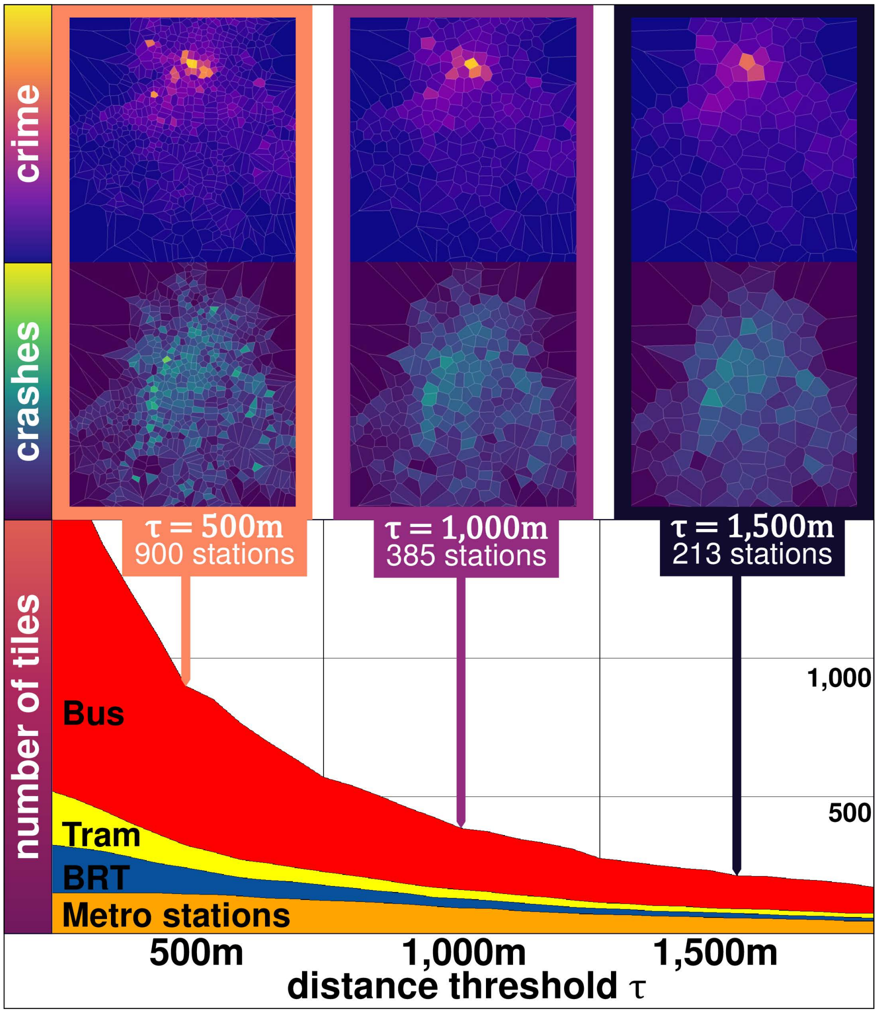

S2 Fig. Effect of the parameter τ. With a smaller τ we obtain more and smaller tiles. The specific underlying pattern of crime and crashes is clearer with smaller thresholds. The panel above shows the number of crimes (top) and crashes (bottom) per unit area for values of $\tau = 500$, $1,000$ and $1,500$ metres.

Other results obtained by modifying the value of τ are also presented here in the Supporting information.

1.5 The impact of physical distance

Physical distance has an impact in terms of the correlation between the heartbeats of each pair of tiles. The distance between each pair of tiles is considered as the Euclidean distance between the corresponding centres. We measure the impact of that distance with a regression between the distance and the pairwise correlation between tiles, which gives us $213 \times 212/2 \approx 22,000$ observations when $\tau = 1,500$ metres, but the number increases considerably as τ decreases and the number of tiles grows, up to nearly 1.5 million observations with $\tau = 250$ metres. Caution needs to be considered, though. When the tiles and the pairwise distances and correlations are constructed, we restrict observations to be at a distance larger than τ and so small distances are not considered with the regression. Therefore, we consider a regression with an intercept equal to 1, so that the regression considers that at small distances, the heartbeat is the same and there is a correlation of 1. Results show that indeed physical distance plays a relevant role (S3 Fig) and the effect is roughly the same varying the values of τ. Still, for most pairs of tiles, the correlation remains high, indicating that in general, there is more crime in the afternoon and evenings and less crime during the night, as well as for road accidents.

1.6 Dealing with the Modifiable areal unit problem

Results show that with different values of τ we get that the number of tiles increase considerably, from 213 tiles with $\tau = 1,500$ metres, up to 1,717 tiles with a value of $\tau = 250$ metres. All tiles have a public transport station as a centre (S3 Fig).

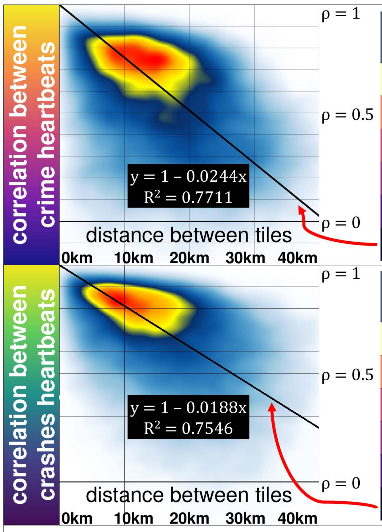

τ	distance coefficient	R^2
250m	-0.0426	0.7867
500m	-0.0359	0.7954
750m	-0.0316	0.7983
1,000m	-0.0283	0.7954
1,250m	-0.0256	0.7797
1,500m	-0.0244	0.7711

τ	distance coefficient	R^2
250m	-0.0361	0.7821
500m	-0.0288	0.7909
750m	-0.0247	0.7917
1,000m	-0.0219	0.7808
1,250m	-0.0198	0.7617
1,500m	-0.0188	0.7546

		maximum distance between stations					
	τ	250m	500m	750m	1,000m	1,250m	1,500m
**numer of tiles**		**1,717**	**900**	**570**	**385**	**276**	**213**
crime	mean per tile	130	247	391	579	807	1,046
	median per tile	91	186	299	412	583	761
	Gini	0.4581	0.4559	0.4574	0.4796	0.5036	0.5057
crashes	mean per tile	547	1,043	1,648	2,440	3,403	4,410
	median per tile	403	832	1,425	2,002	2,924	3,991
	Gini	0.4361	0.3877	0.3749	0.3914	0.4079	0.4131
amenities	mean size per tile	2,863	5,461	8,623	12,766	17,808	23,075
	median size per tile	1,748	3,477	5,808	8,516	11,502	16,585
	Gini	0.5216	0.4954	0.4872	0.5027	0.5189	0.5126
adjusted Rand Index	crime ↔ crashes	0.3819	0.3253	0.3288	0.3085	0.2932	0.4487
	crime ↔ amenities	0.1686	0.2031	0.2573	0.2678	0.2889	0.4687
	crashes ↔ amenities	0.1515	0.1461	0.2252	0.2229	0.2296	0.3492
	cutoff point	0.0016	0.0031	0.0046	0.0072	0.0096	0.0132

S3 Fig. Diffusion of the heartbeats. As distance increases, the top panel shows that the correlation of the crime or the crashes heartbeat between a pair of tiles tends to decrease. The top part shows the physical distance (horizontal axis) between each pair of tiles and the correlation between their crime heartbeat (vertical axis). The line is the result of a regression between both variables, with intercept equal to one. The bottom part shows the physical distance (horizontal axis) and the correlation between the crashes heartbeats for every pair of tiles. The line, as well, represents the regression between physical distance and the correlation, with intercept equal to one. The bottom panel shows the results varying τ and observing a different number of tiles. Number of tiles and Gini coefficient of crime and crashes, and adjusted rand index between the distinct clusterings by varying the size of τ from 250 metres to 1,500 metres.

Notice that there is a spatial concentration of crime as it has been observed before [3]. Here, we consider the number of crimes on each tile and compute the corresponding Gini coefficient of the number of crimes per tile, which gives values from 0.4559 to 0.5057 (both showing a high concentration of crime), which is a "narrow" bandwidth for a specific metric for the concentration of crime [4].

In terms of crashes, we observe also a spatial concentration of the number of crashes per tile, as the Gini coefficient varies from 0.3749 to 0.4361 as we move the dimensions of the spatial units.

Interestingly the amenities are even more concentrated than crime or than crashes. The Gini coefficient of the size of amenities per tile goes from 0.4872 to 0.5216 and for all values of τ, the Gini coefficient is higher for the amenities than it is for the number of crimes or the number of crashes.

Finally, the way we deal with the modifiable areal unit problem is to vary the size of the units and observe that for all cases, with small or large tiles, we obtain similar results. Comparing the adjusted rand index (which is a metric for the level of agreement between two clusterings) gives a number between 0.3085 and 0.4487 for the agreement between the clusterings obtained with crime and crashes; gives between 0.1686 and 0.4687 for the agreement between the clusterings obtained with crime and with amenities; and gives a number between 0.1461 and 0.3492 for the agreement between the clusterings obtained with crashes and amenities. Although the level of agreement varies between the clusterings, results show that for all values of τ they are not the result of randomness and there is some agreement between them.

1.7 Correlation of different moments of the week

There is a high correlation in terms of the distribution of crime and of crashes for the same hour but different days (particularly Monday to Friday). In the case of crime, crosses which go in the direction of time and in the opposite direction are observed, so that the distribution of crime early in the morning is correlated to the distribution later during that day or different days. With smaller values of τ, we obtain more tiles and therefore, more data for each observation (since units are moments of the week and the data is the heartbeat of each tile at that moment). With more tiles (S4 Fig) we get a similar pattern in terms of the daily correlation and crosses for crashes, and also, a similar pattern in terms of the blocks observed for road accidents.

With $\tau = 500$ metres (900 tiles), the observed patterns are similar in terms of the correlations and there is a smaller similarity between moments of the week which do not match (either the same moment on a different day from Monday to Friday, or the opposite moment).

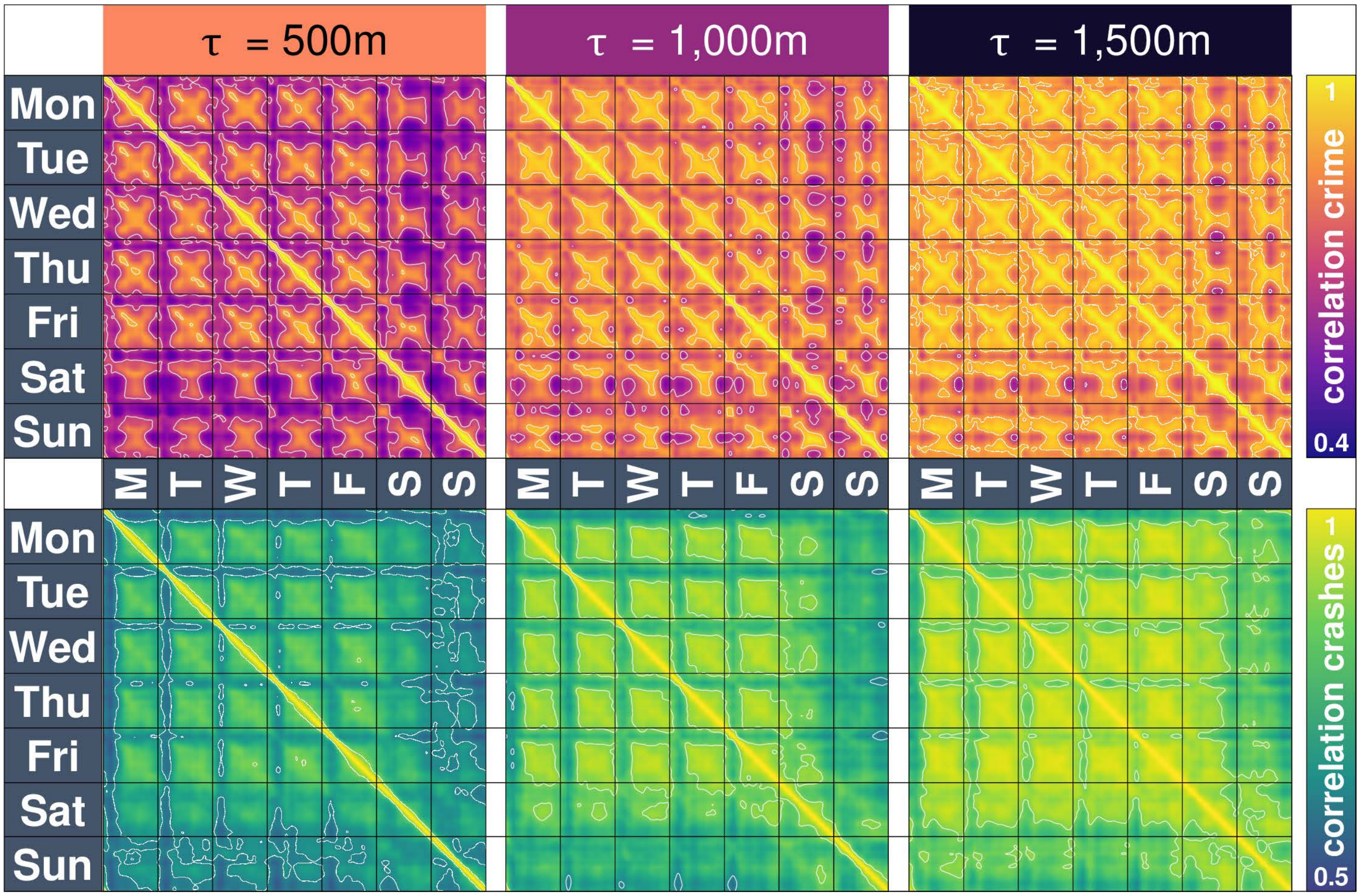

S4 Fig. Correlation between moments of the week. For different values of τ the correlation of the distribution of crime heartbeats and crashes heartbeats per tile shows that from Monday to Friday, moments tend to be similar. In particular, in the case of crime, crosses are observed for all values of τ, which indicate that a Monday morning, for example, has a similar distribution as a Thursday evening. Notice that as τ decreases, the crosses in terms of crime remain almost equally marked, but in terms of crashes, with a smaller τ we also get less correlation, which could be because crashes tend to happen on a highway, for instance, but not necessarily on the exact same spot, and so crashes are grouped for large values of τ but get divided for smaller tiles.

1.8 Classification of amenities

Open access data from the Mexican Economic Census https://www.inegi.org.mx/app/mapa/denue/ gives the location of nearly half a million economic units in Mexico City. Based on their activity, amenities were classified into six broad categories according to their type (S5 Fig).

- **Services** - includes shops, pharmacies, barbers, banks. It represents 66.5% of the amenities in the city and 40.1% of the occupied population.
- **Leisure** - Restaurants, bars, cinemas and other entertainment amenities. It is 12.5% of the amenities and 8.4% of the workforce.
- **Offices** - All types of offices, which represent 8.5% of the amenities and 29.1% of the workforce.
- **Education** - Schools, Colleges and Universities. The size corresponds only to the staff of each institution and it represents 2.6% of the amenities and 7.6% of the workforce.
- **Health** - Hospitals, clinics and laboratories (including veterinaries), with 4.5% of the amenities and 4.7% of the workforce.
- **Manufacture** - All types of factories, with 5.4% of the amenities of the city and 9.5% of the workforce.

The observed amenity mix for each tile is substantially different to other tiles, so that there are tiles with a high number of leisure amenities (bars and restaurants) a tile with a high number of education amenities (mainly the large universities in the city) or tiles with a large number of offices or manufacture. Notice that tiles with a low number of amenities per unit area, since we are considering urban tiles only, are mostly residential or identify tiles such as the Mexico City International Airport, one of the largest urban parks (Bosque de Chapultepec) or other urban structures.

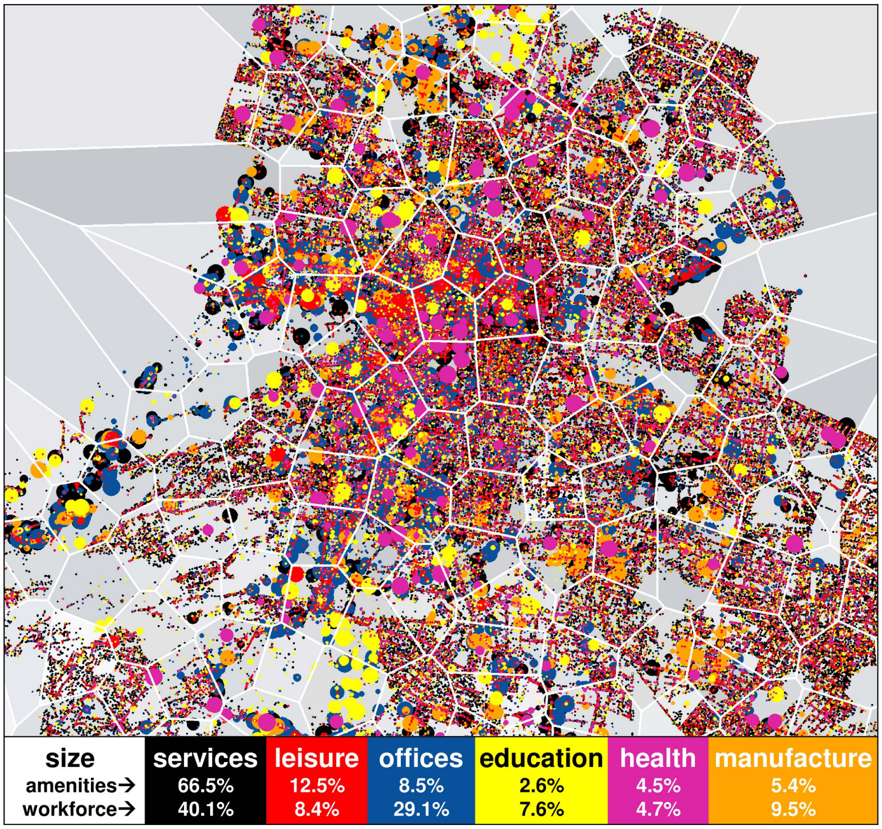

S5 Fig. Amenities per tile. Tiles have a different mixture of amenities. Services, as they include shops and pharmacies, are highly frequent and present in every tile. However, other types of amenities are highly concentrated and there are some tiles with a high level of offices (particularly to the West of the city), some tiles have a high level of education units and the leisure units also seem to be highly concentrated.

1.9 Open access code

The code which can be used to compute the heartbeat of some events with their corresponding time can be found at `https://github.com/rafaelprietocuriel/heartbeat.git`.

References

1. from INEGI OW. Mexican Victimisation Survey ENVIPE, 2018; 2016. `https://www.inegi.org.mx/programas/envipe/2019/`.
2. Silverman BW. Density estimation for statistics and data analysis. vol. 26. CRC press; 1986.
3. Lee Y, Eck JE, O S, Martinez NN. How concentrated is crime at places? A systematic review from 1970 to 2015. Crime Science. 2017;6(1):6.
4. Weisburd D. The Law of Crime Concentration and the Criminology of Place. Criminology. 2015;53(2):133–157. doi:10.1111/1745-9125.12070.
