# Supplementary material for: The heartbeat of the city
Source: PLoS One. 2021 Feb 24;16(2):e0246714. doi: 10.1371/journal.pone.0246714 (PMC7904221; doi:10.1371/journal.pone.0246714)
Supplement: Attachment — Submitted filename: Manuscript PBP.pdf [file pone.0246714.s002.pdf]

Point by point answer

We thank the reviewers for the time to analyse our submission. Based on their comments, we have made some amendments to the manuscript. In the following lines, we explain the specific action taken in response to the reviewers' comments.

Reviewer 1

***Reviewer (R1.1)**: There are several questions that the author should explain or add. Are there any distinction between the tiles in the division of traffic districts?*

The tiles were constructed using the public transport stations, as explained in subsection *Producing city tiles* of the manuscript (page 8). This gives us a more "organic" way to divide the city into non-overlapping regions, as opposed to pre-existing political divisions (for which there is no natural way to refine that division) or neighbourhoods. The procedure depends on a threshold parameter, $\tau > 0$, which is the *maximum distance between stations*, such that if any two stations are at a distance smaller than τ, then only one of them is kept. Then, these tiles are used as units to construct its corresponding crime and crashes heartbeats. Therefore, for a specific value of τ, the stations which are kept are the same and the constructed tiles are the same, both for the analysis of crime and of crashes.

We have added a phrase in page 10, in the subsection *Constructing tile heartbeats* to clarify that the same tiles are used both for crime and for crashes.

***Reviewer (R1.2)**: Is the number of crimes and traffic accidents representative?*

In the case of crashes, our data source is open access data from the Emergency Attention Centre from Mexico City, which combines 911 calls, report calls to emergency buttons, crashes observed through CCTVs, social media and an APP. Repeated calls are usually marked as duplicate incidents and were filtered out. Only confirmed crashes are kept in the database. There are many reasons why most of the serious and lethal crashes should be included in our analysis. The Emergency Attention Centre combines many sources, and since a crash is usually visible to many eyes in the street, it is usually observed and reported. If any attention is needed (such as an ambulance or a police officer) it would be captured by the Emergency Attention Centre system and therefore, issues with respect to under-reported accidents, should not be significant (as observed also by Savolainen et al., 2011).

With respect to crimes, the situation is different. As explained in the manuscript, according to a yearly victimization survey, in Mexico City, only 6% of crimes are reported which induces an unavoidable bias in terms of the victims, the types of crime, whether the criminal was arrested and many more types of bias (ENVIPE, 2018). However, whether a crime happened on a Monday or a Friday and whether it happened at 9:00 or 15:00 should not alter significantly the probability that the victim reports that crime to the police. It is likely that some temporal bias is induced by considering only reported crime, but without any other sources of data to analyse, measure or correct this bias, we assume that the reported crimes reflect the general level of criminal activity in the city at any specific time of the week. Furthermore, we analyse the time in which the

crime happened and not the time in which it was reported to the police. Whilst the time in which the crime is reported to the police should have many issues (since people frequently report their suffered crimes from Monday to Friday, form 8:00 to 19:00) the time in which the crime occurred should reflect at its best the times in which criminals decide to act (1).

In summary, in terms of crashes, our data should reflect most of them (particularly serious and lethal). In terms of crime, we only have 1 in 16 crimes (reported ones) and there is no other source which we could use to expand our analysis. However, in terms of the time in which crimes occur, there should not be any significant bias which alters our results.

In section *Constructing the heartbeats of crime and road accidents*, in the *Data* subsection, we changed a couple of sentences in which the crime and crashes data is presented, to make the issue with our data clearer. Also, in the *Supporting information* section, in the *Cleaning and processing the data* subsection, we added a couple of sentences about the data.

Savolainen, P. T., Mannering, F. L., Lord, D., and Quddus, M. A. (2011). The statistical analysis of highway crash-injury severities: a review and assessment of methodological alternatives. Accident Analysis & Prevention, 43(5):1666–1676.

From INEGI OW. Mexican Victimisation Survey ENVIPE, 2018; 2016.

https://www.inegi.org.mx/programas/envipe/2019/.

(1) See `https://puntodecimal.mx/cdmx/con-las-denuncias-jamas` for a detailed analysis (in Spanish) about the discrepancies between the times in which crime is reported and the time in which crime happens.

Reviewer (R1.3): *What is the correlation and difference between traffic based crime and traffic accident?*

In terms of their location, we observe that some tiles tend to have a higher intensity of both, crimes and crashes, although we have not analysed traffic-based crimes separately (such as car thefts) but the spatial correlation is not high. There are some tiles (as observed in Figure 12 with smaller tiles) which have a high intensity of crimes and low intensity of crashes.

Since one of the reason why there is a high intensity of crimes or of crashes on certain tiles could be related to a high flow of individuals, then it is not surprising that central tiles are, in general, high on both events, whereas peripheral tiles have lower intensities. However, since we are dealing with two distinct types of events, one in which there is an intention and an objective (a crime) and one which is the result of an accident (a crash) we have treated both events separately in the manuscript.

There are some interesting correlations between both events. For example, Beland and Brent analysed abnormally bad traffic in Los Angeles and noticed that it could increase the incidence of domestic violence (and not of any other type of crime), whereas Ando and others showed that in Toyota City, a Japanese city, there could be some common factors, such as narrow streets, affecting the occurrence of traffic accidents and urban crime. However, since our focus is robberies (and not traffic-based crimes), further investigation of the link between road accidents and traffic-based crime is beyond the scope of our manuscript and so these references are not included in our submission

Beland, L.-P. and Brent, D. A. (2018). Traffic and crime. Journal of Public Economics, 160:96–116.

Ando, R., Higuchi, K., and Mimura, Y. (2018). Data analysis on traffic accident and urban crime: a case study in Toyota City. International Journal of Transportation Science and Technology, 7(2):103–113.

Reviewer (R1.4): *In Figure 10, what is the basis for the correlation coefficient to take 0.4 and 0.5? What's the point?*

The time correlations observed in crime moments (top panel of figure 10) range between 0.43 and 1, whereas the correlations between crashes moments (bottom panel) range between 0.52 and 1. Both panels could use a range between 0.4 and 1, but it would mean that the colours at the bottom of the crashes correlations would simply not appear in the figure.

In general, the fact that the range from the crashes correlations is slightly higher than it is for crime means that some specific pairs of times of the week (for instance, Saturday evenings and Monday nights in the case of crime, which is when the lowest correlation is observed) there is a more pronounced change for crimes that there is for crashes (for instance, Sunday nights and Monday mornings, which is also when the lowest correlation is observed in the case of crashes moments).

We added a sentence in the caption of the figure to highlight that both panels use a different range and its reasons.

Reviewer (R1.5): *For the statistical results, the specific figures shall be reflected and the charts shall be combined.*

Thank you for your suggestion. We have merged figures 13 and 16 into a single figure which reflects the statistical analysis as the distance threshold τ varies. Therefore, we have made also the corresponding changes in the manuscript.

Thank you for your suggestions to improve our manuscript.

Reviewer 2

***Reviewer (R2.1)**: The authors are recommended to conduct a more comprehensive literature review work. The introduction section did not clearly demonstrate the unique contributions of this study over the existing studies. Moreover, the clear definition of the problem of this study is missing.*

We thank the reviewer for their comments. We have changed the structure of the introduction to reflect our research questions and to show the contributions of our study and the gaps we have identified in the literature. Previous research has focused on temporal aspects about crime and crashes, but time is generally analysed as a sequence and the cyclic aspect of time is usually considered as an autorregresive part of a time series. However, whether an event (crime or crash) takes place on a Monday morning or a Tuesday night or the weekend alters conditions completely, and so we based our analysis on the cyclic repetitions given by weekly time units. Our main contribution stems from the idea to overlap weeks and observe time through cycles, so that if a crime, for example, happens on a Friday night, it is considered to occur under similar conditions to other events which also happened on a Friday night, even if they are months or even years apart. This allowed us to capture repeating weekly patterns of crime and crashes. In particular, our novel contributions are grouped below in three distinct areas.

Firstly, we identified weekly units of time as a relevant way to analyse social events. Being weekly units of time cyclic, we analyse the correspondence between the same moments of the week, but on different weeks (for instance, Tuesdays at 21:00). We constructed a new mathematical function, the *heartbeat* as the continuous intensity of discrete events, based on a modified kernel density estimation.

Secondly, as a case study, we computed the heartbeats of crime and of crashes at a city level in Mexico City and detected have distinct heartbeats, but are someway synchronised with peaks and valleys throughout the week.

Thirdly, we designed a novel way to divide the city into distinct tiles based on public transport stations and a distance parameter τ, which gives us a more organic way to divide the city and provides certain flexibility, as we can reduce or increase the size of the tiles. Crime and crashes heartbeats are constructed at a tile level and we prove that heartbeats have three valuable properties: are different, statistically speaking; nearby tiles tend to have more similar heartbeats than distant tiles; and finally, land use, analysed by the amenity mix of the tiles, is shown to be related to the shape of the heartbeats.

Based on your suggestion, we have made some changes to the structure of our manuscript, particularly in the introduction, to highlight our research questions, and our unique contributions. We have added some subsections in the introduction to help readers go through the document easily, taking into account that our research has many components. Also, we modified the structure of the literature review section, to highlight what is known and what is not fully understood with respect to crime and crashes, which our research aimed at answering. It is indeed difficult to capture such complex and broad social processes as crime and crashes and try to summarise them into a single introduction and literature review sections. We have added some references to a couple of review papers, trying to keep our introduction and literature review sections succinct but not too superficial. In particular, we have added the paper by D'Orsogna and Perc

(2015) and the paper by Gordon (2010) for a review in terms of crime and complexity. Further, in terms of crashes, we added the review paper by Savolainen and others (2011). Thank you for your suggestions to improve our manuscript.

D'Orsogna MR, Perc M. Statistical physics of crime: A review. Physics of Life Reviews. 2015;12:1–21.

Gordon MB. A random walk in the literature on criminality: A partial and critical view on some statistical analyses and modelling approaches. European Journal of Applied Mathematics. 2010;21(4-5):283–306.

Savolainen, P. T., Mannering, F. L., Lord, D., and Quddus, M. A. (2011). The statistical analysis of highway crash-injury severities: a review and assessment of methodological alternatives. Accident Analysis & Prevention, 43(5):1666–1676
